# Alterations in Fixation Indices in Primary Open-Angle Glaucoma by Microperimetry

**DOI:** 10.3390/jcm11092368

**Published:** 2022-04-23

**Authors:** Katarzyna Zabel, Przemyslaw Zabel, Karolina Suwala, Aleksandra Gorczyca, Damian Jaworski, Martyna Kaluzna, Martyna Gebska-Toloczko, Kacper Wnuk, Katarzyna Buszko, Jakub J. Kaluzny

**Affiliations:** 1 Department of Sensory Organ Studies, Collegium Medicum, Nicolaus Copernicus University, 85-830 Bydgoszcz, Poland; przemo.zab@gmail.com (P.Z.); karolinasuwala4@gmail.com (K.S.); agorczyca@doktorant.umk.pl (A.G.); martyna.gebska@gmail.com (M.G.-T.); jjkaluzny@oftalmika.pl (J.J.K.); 2 Division of Ophthalmology and Optometry, Department of Ophthalmology, Collegium Medicum, Nicolaus Copernicus University, 85-168 Bydgoszcz, Poland; jaworski.damian94@gmail.com (D.J.); martynakaluzna93@gmail.com (M.K.); 3 Department of Biostatistics and Biomedical Systems Theory, Collegium Medicum, Nicolaus Copernicus University, 85-067 Bydgoszcz, Poland; kcpr.wnuk@gmail.com (K.W.); buszko@cm.umk.pl (K.B.)

**Keywords:** primary open-angle glaucoma (POAG), fixation stability, microperimetry (MP), retinal function, retinal structure

## Abstract

The aim of this study was to determine whether primary open-angle glaucoma (POAG) is associated with changes in fixation stability parameters assessed by microperimetry (MP) and whether the severity of glaucoma is related to a deterioration in these indicators. This study analyzed fixation stability using MP macular analyzer integrity assessment (MAIA) in patients with mild and moderate/severe POAG and healthy controls. The resulting fixation indices were correlated with parameters used to assess retinal function with MP and standard automated perimetry (SAP) and retinal structure with optical coherence tomography (OCT) and OCT angiography (OCTA). We enrolled 54 eyes in the POAG groups (32 eyes with mild POAG and 22 eyes with moderate/severe POAG) and 24 eyes in the healthy group. It was shown that fixation stability in POAG eyes deteriorated with increasing disease severity, and significant differences in bivariate contour ellipse area (BCEA) including 95% of fixation points were observed among groups (*p* = 0.042). Quantitative analysis of structural and functional retinal parameters also showed significant deterioration with the progression of glaucoma (*p* < 0.001). Correlations among fixation parameters and abnormalities in the retinal structure and function were confirmed. We concluded that POAG is associated with disturbances in the fixation pattern, which worsen as the disease progresses and can be effectively assessed by performing a MP test.

## 1. Introduction

Primary open-angle glaucoma (POAG) is a chronic progressive optic neuropathy, leading to the degeneration of retinal ganglion cells, typical changes in optic disc morphology, and characteristic visual field (VF) defects [1]. Although the first signs of the disease are associated with retinal nerve fiber layer (RNFL) damage in the peripheral part of the retina, previous studies have revealed structural and functional abnormalities in the central part of the macula [2]. Best-corrected visual acuity (BCVA), the main functional parameter used to assess central vision loss, is usually preserved in patients with POAG, despite a significant reduction in retinal sensitivity [3]. To better understand this discrepancy, fixation behavior should be examined as an additional parameter for assessing central macular function [4].

The macular sensitivity of the retina and fixation behavior can be assessed using microperimetry (MP). The MP technique uses continuous infrared scanning laser ophthalmoscope (SLO) imaging to track the retina and assess macular function. Fundus imaging with retinal landmark tracking enables precise correlation of macular anatomy with light sensitivity in MP. In addition, it is possible to control a patient’s fixation independent of eye movement and to correct gaze movements. Fixation stability is an objective test performed by tracking retinal landmarks then plotting the scatter of a cloud of fixations points (CFP) on a retinal image reference map. A normal emmetropic subject fixating on a point at optical infinity will exhibit some retinal movement. This is due to involuntary eye movements such as physiological nystagmus, drift, as well as microsaccades as corrective movements to compensate for involuntary head movements [5]. Fixational eye movements have a large influence on visual perception. Ocular drift transforms visual stimuli in a way that increases spatial acuity. Microsaccades improve vision by displacing the fovea and stimulating many photoreceptors. The spatial distribution of these fixation points is related to the stability of fixation: a small fixation area is associated with a more stable fixation compared with a larger area. In healthy subjects using central fixation, the size of these eye movements is small, and all fixations fall within a few minutes of the arc of the target center. This ability to maintain stable fixation is impaired in people with eye diseases and diseases affecting the central nervous system [6,7,8,9,10].

Alterations in fixation pattern in glaucoma are not well understood. Only a small number of studies have been reported; however, the results indicate the possibility of disturbances in the stability of fixations in eyes with glaucoma [11,12,13]. To the authors’ knowledge, no studies have compared the fixation patterns in patients with POAG at different stages of disease advancement with those of a healthy control group. Moreover, correlations among disturbances in the structure/function of the retina and changes in fixation stability have not been analyzed. Information on the fixation pattern could make a significant contribution by not only deepening the knowledge of the pathophysiology of POAG, but, in the future, may also become a biomarker used in diagnosis and for monitoring the severity of glaucoma.

The aim of this study was to determine whether POAG is associated with changes in fixation stability parameters assessed by MP and whether the severity of glaucoma is related to the deterioration in these indicators. Additionally, we analyzed correlations among fixation indices and retinal function/structure parameters assessed by MP, standard automated perimetry (SAP), optical coherence tomography (OCT), and OCT angiography (OCTA) in eyes with mild and moderate/severe POAG and healthy controls.

## 2. Materials and Methods

### 2.1. Study Design and Patient Recruitment

In this cross-sectional study, all subjects were recruited and assessed at the Oftalmika Eye Hospital (Bydgoszcz, Poland) between 2019 and 2020. The study was performed according to the tenets of the Declaration of Helsinki. All participants provided written informed consent prior to their inclusion in the study. The study protocol was approved by the Bioethical Commission of Nicolaus Copernicus University in Torun, Collegium Medicum in Bydgoszcz (approval number 600/2019).

All participants underwent a detailed ophthalmological examination, including measurement of the following parameters: refractive error (Topcon KR-890, Tokyo, Japan), BCVA, slit-lamp biomicroscopy with gonioscopy and dilated fundus examination using a Volk lens, intraocular pressure (IOP; Icare TAO1 i, Finland Oy, Vantaa, Finland), pachymetry (Tomey EM-3000, Tomey Corporation, Nagoya, Japan), and axial length measurement (IOL Master 500, Zeiss Humphrey, Dublin, CA, USA). In addition, patients were examined using a Spectralis OCT (Heidelberg Engineering, Heidelberg, Germany), OCTA (Optovue, Inc., Fremont, CA, USA), Humphrey Field Analyzer II (HFA; Carl Zeiss Meditec, Dublin, CA, USA), and macular analyzer integrity assessment (MAIA) MP (Centervue, Padova, Italy). All examinations were performed in one day by the same ophthalmologist.

Patients treated for perimetric glaucoma for at least 6 months were included in the study. All participants were required to have POAG and meet the following criteria: the presence of features of glaucomatous optic neuropathy accompanied by a decrease in peripapillary RNFL (pRNFL) thickness corresponding to VF loss in SAP with a normal anterior chamber and open angle based on slit-lamp and gonioscopic examinations, respectively. Glaucomatous disc changes were defined as a vertical cup-to-disc ratio greater than 0.7, asymmetry greater than 0.2 between the two eyes, optic disc hemorrhage, and neuroretinal rim changes consisting of pallor or localized notching in the absence of any other ocular or neurological pathology. Glaucomatous VF losses were identified by static perimetry using a threshold approach. One of the following changes observed in two consecutive visual field tests were used as a criterion for glaucomatous damage: a cluster of three or more adjacent points in a typical localization for glaucoma with a *p*-value less than 0.05 in pattern standard deviation (PSD) and one point with a *p*-value less than 0.01 in PSD, a glaucoma hemifield test result outside normal limits, and/or an average PSD value calculated for the entire tested area in less than 5% of healthy eyes. Patients with POAG were further classified into two groups based on the severity of VF damage in SAP: mild glaucoma was defined as a VF mean deviation (MD) greater than −6 dB; moderate/severe glaucoma was defined as an MD less than −6 dB.

Age- and sex-matched subjects with no ocular or neurological pathology and normal VF in SAP were taken as controls.

The general exclusion criteria were: BCVA less than 0.6, refractive error above ±3.0 Dsph, IOP less than 23 mmHg, any media opacity, ophthalmic surgery, except for uncomplicated phacoemulsification cataract and uncomplicated anti-glaucoma surgery if at least six months had passed since the surgery. People with vascular or nonvascular retinopathies, ocular or systemic diseases known to impair VF, nonglaucomatous optic neuropathies, and macular pathology were also excluded.

### 2.2. Microperimetry

The MAIA MP technique combines SLO, static perimetry, and fundus imaging. The mechanism of observation is an infrared superluminescent diode with a wavelength of 850 nm, which provides high-quality images, even with pupil diameters of 2.5 mm. The maximum level of illumination is 318.47 cd/m^2^. The light appears in ranges of attenuation from 0 to 36 dB in 1 dB steps. The background luminance is 1.27 cd/m^2^. Goldmann-type size III stimuli were presented for a duration of 200 ms. MP delivers information in the form of retinal threshold sensitivity and fixation stability. The average sensitivity threshold (average threshold—AT) in dB was measured using the expert exam option with a MAIA standard macular grid pattern (37 stimulus points) over 10 retinal degrees (±5 degrees around the macula). The standard 4-2 projection strategy was used, and all measurements were performed monocularly in a darkened room. During the examination, patients were instructed to look at the center of the fixation target with correct operation of the response button when stimuli was shown. Eye movements were registered by an integrated eye-tracker system with a frequency of 25 Hz, with the dual purpose of correcting ocular misalignment and registering the subjects’ fixation pattern. The MP system can describe fixation stability in two ways. The first method calculates the percentage of fixation points (PFP) within a circle of 1° and 2° radii (defined as P1 and P2, respectively) centered in the barycenter of the CFP. The second method measures the bivariate contour ellipse area (BCEA), which is the macular area surrounding all fixation movements within 1 or 2 standard deviations, consequently including 95% (BCEA95) and 63% (BCEA63) of fixation points. This analysis also considers the major and minor axes, which are two orthogonal diameters describing the range of fixation points. The BCEA orientation is the angle between the ellipse major axis (EMA) and the horizontal axis (HA) of the visual meridian, with values between 0° and +90° corresponding to angles measured counterclockwise between the HA and EMA; values between 0° and −90° corresponding to angles measured clockwise between the HA and EMA; 0° corresponding to a horizontal orientation and 90° corresponding to a vertical orientation.

Currently, the clinical interpretation of the MAIA results is based on the manufacturer’s analysis printout. The printout reports the measured retinal sensitivity at each test loci (pointwise sensitivity), as well as the mean sensitivity across all test loci. Retinal sensitivity indexes AT and Macular Integrity are shown in a color chart where green, yellow, and red, respectively, represent normal, suspect, or abnormal sensitivity. The analysis printout also includes a “histogram of thresholds frequencies”, which shows the distribution of retinal sensitivity compared to the Gaussian distribution of a “normal reference” of healthy eyes between 20 and 80 years of age. Furthermore, the analysis printout reports a fixation plot with numerical values for BCEA63 and BCEA95 expressed as the ratio of the EMA and HA, their area in square degrees, and angle. On the printout, there is also a scale of fixation stability based on P1 and P2 values, where green, yellow, and red mean stable, relatively stable, and unstable fixation, respectively. A graph with the duration of the test is also displayed.

### 2.3. Optical Coherence Tomography

All patients underwent OCT imaging with the objective of measuring the thickness of the pRNFL and ganglion cell complex (GCC). The pRNFL thickness in μm mean globally (G), and the defined quadrants, superior (S), inferior (I), temporal (T), nasal (N), were analyzed over 360° using a Spectralis OCT device (Heidelberg Engineering, Heidelberg, Germany). Each circular scan consisted of 768 A-scans. The scanned circle was 3.46 mm in diameter and cocentered with the optic nerve head (ONH). A built-in Avanti RTVue XR (Optovue, Inc., Fremont, CA, USA) glaucoma module was used to measure the thickness of the retinal GCC. The GCC scan was centered 1 mm temporal to the fovea and covered a circular macular area of 6 mm in diameter. The global GCC thickness and the thickness of the superior and inferior hemifields in μm was assessed. Furthermore, all scans had to fulfil consensus criteria for retinal OCT quality assessment (OSCAR-IB) to ensure comparability and quality of OCT images [14].

### 2.4. Standard Automated Perimetry

VF examinations were performed with a 24-2 VF test grid using an HFA II with a standard Swedish interactive threshold algorithm (SITA). The 24-2 VF test measured retinal sensitivity thresholds at 24 degrees temporally and 30 degrees nasally, including 52 test points separated by 6 degrees (excluding two blind spot locations). A Goldmann-type size III stimulus with a background luminance of 31.5 asb was used. Near correction was provided as needed. Only reliable VF tests (fixation loss less than 33%, false-positive and false-negative rates less than 10%) without artifacts and cases with no evidence that the abnormal results were caused by diseases other than glaucoma were included. Global indices to assess glaucomatous defects MD, PSD, and visual field index (VFI) were evaluated for the statistical analysis. MD (in dB) is the average of the differences from the mean of age-adjusted normal values of all visual field locations tested. The severity of glaucoma was recorded as a MD value. PSD (in dB) is an indication of any local abnormalities in an individual’s visual field calculated by the device. The VFI is a trend analysis that scales the overall visual field status from 100% (normal) to 0% (end-stage glaucoma).

### 2.5. Optical Coherence Tomography Angiography

OCTA imaging was carried out using an Avanti RTVue XR with AngioVue software (version 2017.1.0.151), which provides noninvasive qualitative visualization and quantitative assessment of the retinal vascular network using the split-spectrum amplitude-decorrelation angiography (SSADA) algorithm. We considered the obtained OCTA results as a structural parameter due to the fact that, after appropriate segmentation, only the density of the microvessel network is analyzed, not the blood flow. The OCTA device can perform 70,000 A-scans per second and achieves measurements with an axial resolution of 5 μm using a light source with a wavelength of 840 ± 10 nm and a bandwidth of 45 nm. To correct for motion artifacts, the device is equipped with DualTrac Motion Correction technology, and orthogonal fast-scan directions (horizontal and vertical) are combined. The AngioVue software is equipped with three-dimensional Projection Artifact Removal (3D PAR), which reduces projection artifacts in deeper retinal layers while maintaining their authentic layout. The macula was analyzed using B-scans covering an area of 6 × 6 mm^2^ on an area of 4.5 × 4.5 mm^2^ centered on the ONH, and peripapillary vessels were analyzed. The images consisted of two sets of B-scans repeated horizontally and vertically, each consisting of 400 A-scans. Data analyses including automatic segmentation of the superficial vascular plexus (SVP) and deep vascular plexus (DVP) of the macula and the peripapillary radial peripapillary capillary (pRPC) layer in the ONH area were performed on commercially available software and expressed in %. The en face OCTA image is a grayscale image composed of a range of pixels from black with a value of 0 to white with a value of 255. Each of the thresholding strategies uses a different approach to define the level above which pixels are white and below which pixels are black. Using these tools, images are converted from grayscale images to binary images, and vessel density is calculated as the ratio of the area occupied by the vessels divided by the total area. In ONH scans, vessel density was analyzed in the peripapillary area, which extends outward from the ONH border with an elliptical area between 2–4 mm. The RPC layer was defined as extending from the inner limiting membrane (ILM) to the posterior border of the RNFL. In the macula, vessel density was analyzed on the entire surface of the 6 × 6 mm^2^ en face images. The SVP is comprised of the area between the ILM and the outer boundary of the inner plexiform layer (IPL), whereas the DVP is comprised of the area between the outer boundary of the IPL and the outer boundary of the outer plexiform layer (OPL). Both eyes of each patient were examined after pupil dilation on the same day between 1:00 and 4:00 PM. Only measurements of good technical quality with a signal quality (SQ) of 6 or more on a 10-degree scale, with which the commercial camera was equipped, qualified for further analysis. Measurements with motion artifacts on the en face images (irregular patterns of vessels or a blurred ONH boundary of the) were rejected.

### 2.6. Statistical Analysis

All statistical analyses were performed in R (version 3.6.2.). Prior to the study, our goal was to determine the sample size required to compare BCEA95 values (parameter considered as primary endpoint) between healthy and POAG patients with a test power of >80% and α = 0.05; however, there are no data available regarding the mean and variance of BCEA95 for POAG patients. Therefore, we decided to conduct a small pilot study (n1 = n2 = 15 for both healthy and POAG patients) in order to determine the sample size. The mean value of BCEA95 for healthy patients in the pilot study was 2.10, and for POAG patients—4.31. The standard deviation was 3.05. We determined the sample size based on pilot study results in order to obtain >80% power of a test with α = 0.05, a sample size of at least 25 for each group was required + additional 20% to take account for possible dropouts. In the end, 77 eyes were included in the study. Due to absence of a normal distribution (as assessed by Shapiro–Wilk test), all continuous variables were analyzed using nonparametric tests. Differences between group means were assessed using the Kruskal–Wallis test or χ^2^ test for continuous and categorical variables, respectively. Spearman’s rank correlation coefficient was calculated to determine the correlation among fixation indices and retinal function parameters. A two-sided *p*-value less than 0.05 was considered statistically significant.

## 3. Results

Initially, 56 eyes of 32 POAG patients and 24 eyes of the 16 participants of the healthy group who met the outlined inclusion and exclusion criteria qualified for the study. Of these eyes, 2 with POAG (3.5%) and 1 healthy eye (2.4%) were subsequently excluded due to poor-quality imaging tests or unreliable VF test results. A total of 54 eyes from 31 POAG subjects were included in the final analysis. Based on the degree of VF loss in SAP, 32 eyes of patients with POAG were assigned to the mild POAG group (MD −2.57 ± 1.54 dB) and 22 eyes were included in the moderate/severe POAG group (MD −12.77 ± 7.67 dB). The studied groups did not differ in age (*p* = 0.522) or sex (*p* = 0.932). There were no significant differences among groups in terms of BCVA, IOP, AL, CCT, and MP examination time (*p* > 0.05). Demographic and clinical characteristics of the study participants are summarized in Table 1.

### 3.1. Structural and Functional Data

Quantitative analysis of structural (GCC and pRNFL thickness, SVP and pRPC vessel density) and functional (VFI, MD, PSD, AT) retinal parameters showed significant deterioration with the progression of glaucoma (*p* < 0.001). Although eyes with glaucoma showed reduced vessel density at DVP, these differences were not significant among study groups (*p* = 0.1641). The ratio of DVP to SVP whole density was 0.99 in the healthy group, which differed significantly from values obtained in the mild and moderate/severe POAG groups, with index increases of 1.11 and 1.21, respectively (*p* < 0.001). Comparisons of structural and functional characteristics are presented in Table 2.

### 3.2. Fixation Indices

**Figure 1:**
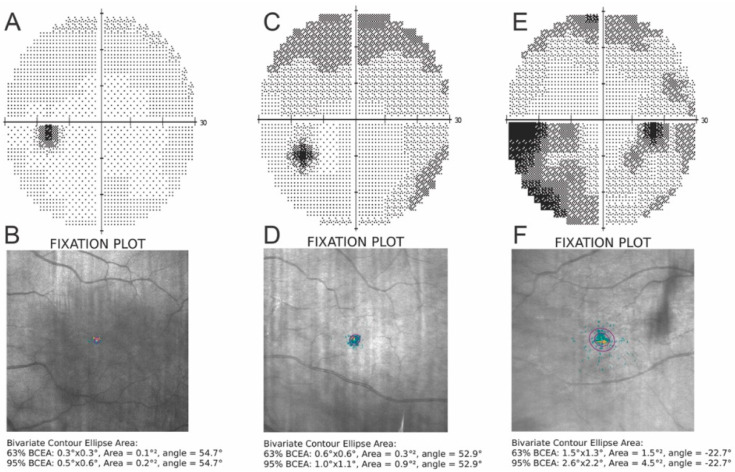
The figures show changes in the visual field and fixation pattern in a healthy control eye (**A**,**B**), respectively, in the eye with mild POAG (**C**,**D**), respectively, and in the eye with moderate/severe POAG (**E**,**F**), respectively. As the severity of glaucoma progresses, the fixation parameters deteriorate, as shown in the figure.

Fixation parameters were analyzed in each group. Although the P1 and P2 indices deteriorated with increasing POAG severity, differences between groups were not statistically significant (*p* > 0.05). Both BCEA63 and BCEA95 values increased with the degree of POAG. However, of the fixation indices analyzed, only BCEA95 was statistically significantly different between study groups (*p* = 0.042). The difference in BCEA63 values was almost statistically significant (*p* = 0.051). The diameters describing the extent of the fixation area along the horizontal and vertical axes (H95 and V95) increased with severity of the disease (Figure 1). However, the analysis revealed no statistically significant differences among study groups (*p*> 0.05). In the POAG and healthy groups, the ratio of H95 to V95 was close to one and the fixation ellipse was close to a circle. Results of the comparative analysis are presented in Table 3.

When analyzing the relationships between fixation indices and global structural parameters, we found a significant correlation of P1, BCEA63, BCEA95, and H95 only with global GCC thickness and SVP vessel density. In analyzing the relationship between fixation indices and regional structural parameters, the strongest correlations were found with pRNFL thickness in the temporal and superior quadrants, and GCC thickness in the superior and inferior hemifields. The results are presented in Table 4.

In 77 eyes, analysis of the relationships between fixation indices and retinal function parameters showed significant correlations between all fixation parameters and AT, assessed by MP. There was also a weak but significant correlation between BCEA (95 and 63) and VFI, assessed by SAP, and between BCEA63 and BCVA. The fixation index BCEA95 exhibited the strongest correlations with both structural and functional parameters of the retina. The results are reported in Table 5.

## 4. Discussion

This study analyzed fixation stability using MP MAIA in patients with mild and moderate/severe glaucoma and healthy controls. The resulting fixation indices were correlated with parameters used to assess retinal function with MP and SAP and retinal structure with OCT and OCTA. Fixation stability in glaucoma patients deteriorated with increasing disease severity and significant differences in BCEA95 were observed among groups. The correlation among fixation parameters and abnormalities in retinal structure and function were also confirmed.

MP is a modern method that allows detailed analysis of the macular function based on direct correlation with anatomical aspects of the retina observed using an eye tracker. Recently, fixation pattern analysis has attracted increasing interest from researchers as an objective method for studying the function of the visual system. Fixation is recorded during standard MP tests (dynamic fixation) performed to assess retinal threshold sensitivity but can also be recorded as an isolated fixation task (static fixation) [15,16]. In our study, we evaluated dynamic fixation during retinal sensitivity testing. The MP software automatically analyzed the fixation stability using two different methods: the clinical classification proposed by Fujii et al. and BCEA analysis [17,18]. The main advantage of the method of Fujii et al. is the clinically relevant classification of fixation stability: Eyes with a P1 value greater than 75% are classified as having stable fixation. If P1 is less than 75% and P2 is greater than 75%, fixation is classified as relatively unstable; if both P1 and P2 are less than 75%, the pattern is described as unstable fixation [17]. However, this method has come under scrutiny in the literature due to the arbitrarily selected fixed circular area of 1° and 2° in radius used to determine the stability index [19]. The BCEA analysis method for evaluating fixation stability in MP proposed by Crossland et al. calculates the area and orientation of an ellipse encompassing a specific portion of the fixation point dataset. The advantage of the BCEA calculation is that it is based on a mathematical model used to describe the movement of variables in statistics; however, the BCEA is not associated with any clinical classification in MP [20,21].

Our results support previous reports on the suitability of MP for assessing fixation behavior, especially when the BCEA is analyzed. In 2016, Morales et al. published a study in which a clinical reference database for fixation stability metrics measured by MP MAIA was established, based on measurements obtained from 358 healthy volunteers. Average values of 0.80°^2^ for the BCEA63 index and 2.40°^2^ for the BCEA95 index were obtained. The mean values of P1 and P2 were 95% and 99%, respectively [19]. These values are consistent with the fixation indices measured in our healthy group. Importantly, our analysis showed significant differences in fixation stability among study groups when described by BCEA95. Despite deterioration in the P1 and P2 indices with increasing severity of glaucoma, we did not show any statistical significance. Our results confirm those of Longhin et al., which concluded that BCEA analysis results in a greater accuracy in detecting minimal quantitative changes in fixation stability, compared with the standard clinical classification [22]. Moreover, a strong relationship was previously demonstrated between fixation stability, as measured by BCEA, and a very weak correlation between P1 and P2 and many reading ability parameters used to assess visually impaired patients, which demonstrates the advantage of the fixation stability based on BCEA rather than the Fujii classification system [23].

In our study, we demonstrated the usefulness of fixation stability assessment, confirmed by correlations among the disruption of retinal structure/function and changes in fixation stability parameters in the study groups. The strongest correlation was observed between AT of the retina and BCEA indices. In a previous study, Shi et al. compared patients with early and moderate stages of glaucoma to healthy controls and analyzed fixation data from microperimetric tests using MP-1 (NIDEK Technologies, Vigonza, Italy). Significant differences in fixation stability were observed among groups if the PFP was maintained within the central 2° circle. A relationship between fixation stability within the central 2° (P1 in MAIA microperimeter, respectively) and AT of the retina was also observed in the POAG group. However, in this work, the assessment of fixation indicators did not include BCEA analysis [12]. The reduced retinal sensitivity in MP associated with alterations in microcirculation in glaucomatous eyes was previously investigated. The correlation between decreasing AT in MP and microvascular network damage in macular SVP in all patients with POAG was demonstrated. This relationship was stronger than the correlation between pRNFL thickness measurements and VF parameters in SAP [3]. Additionally, our current study showed a significant correlation between fixation indices and vessel density in SVP assessed by OCTA.

Montesano et al. also reported differences in fixation patterns in patients with glaucoma and the healthy control group. Fixation stability was measured as BCEA using two novel metrics: mean Euclidean distance (MED) and sequential Euclidean distance (SED). These measures were designed to capture the spread of fixation points and the frequency of position changes during fixation, respectively. The authors reported subtle changes in fixation more accurately described by features of the temporal sequence displacements (SED), rather than measures of fixation spread such as BCEA. They suggest that glaucoma patients try to enhance perception of the fixation target by frequent shifting between different positions. Moreover, it was observed that SED has a significant association with MD, but neither BCEA nor MED were significantly correlated [11].

We found that the horizontal (H95) and vertical (V95) axes increased with the severity of glaucoma in relation to the healthy group. However, despite the larger area, the BCEA remained circular in all POAG groups. An analysis of major BCEA axis values was performed by Gil-Casas et al. using MP in subjects with multiple sclerosis (MS), with and without previous optic neuritis (ON). They found that the shape and size of the fixation ellipse differed among groups. The sizes of the axes (H95 and V95) were similar in the control group, and the BCEA was circular in shape. In the MS group without a history of ON, the size of both axes increased compared with the control group. In the MS group with previous ON and contralateral eyes without ON, values of BCEA increased more on the vertical axis than on the horizontal axis, resulting in a vertical ellipse [24]. Changes in fixation pattern, including the ellipse shape and size, described in neurodegenerative diseases may be different, which may have prognostic and diagnostic significance. Post-mortem and in vivo studies conducted to date have shown that neurodegenerative disorders are characterized by a loss of specific neuron populations. In glaucoma, vision loss and dysfunction are the result of retinal ganglion cell death, atrophy, and axon degeneration extending to central visual targets in the brain [25,26]. Glaucomatous damage has an impact on vision and oculomotor structures in the brain, including the lateral geniculate nucleus and visual cortex [27]. Dysfunction in oculomotor control has also been described in various neurodegenerative disorders (e.g., Alzheimer’s disease, parkinsonian disorders, MS) [28]. Fixation is a dynamic process that is actively controlled by neuronal mechanisms localized in the cerebrum, brainstem, and cerebellum. During fixation, eyes continue to move with a combination of microsaccades, smooth ocular drifts, and tremors. Microsaccades are the largest of these fixational eye movements. Abnormal eye movement, fixation instability, and foveation are likely a sign of inflammatory and neurodegenerative effects on the optic nerve and the widespread network of central afferent and efferent visual and oculomotor control pathways that are necessary for normal fixation [5,28,29]. These theories are supported by our results that in glaucoma, as in other neurodegenerative diseases, fixation disorders occur.

There are several limitations to this study. Due to relatively small sample size, the results might not be completely representative of the general population. For this reason, both eyes were included in the analyses when the study criteria were met. To our advantage, the evaluation of the results showed that in such cases, when eyes were at different stages of glaucoma advancement, fixation parameters also differed and were worse in the eye with more advanced glaucoma, based on the VF in SAP. Patients with POAG did not discontinue ocular hypotensive eye drops, which might affect ocular blood flow. The effect of antihypertensive eye drops is likely to persist for 1–4 weeks from the time of withdrawal; therefore, for ethical and medical reasons, patients with POAG involved in the present study did not stop using them. For the same reasons, when patients from both study groups were taking systemic drugs, their use was not discontinued during the study. Nevertheless, it seems that these topical and systemic drugs should not affect the obtained fixation parameters [30,31,32].

## 5. Conclusions

In summary, this was the first study to analyze fixation parameters obtained using MP MAIA and to correlate them with structural and functional changes in the retina in patients with mild POAG, moderate/severe POAG, and healthy controls. The results show that POAG is associated with disturbances in the fixation pattern, which worsen as the disease progresses and can be effectively assessed by performing a MP test. We demonstrated that changes in fixation indices correlate with structural changes in OCT and OCTA, as well as functional changes in MP and SAP. The observed alterations in fixation are probably a way of adapting visual perception to operating under conditions of reduced RGC. Due to the stimulation of photoreceptors in a larger area of the retina and, therefore, more RGCs, the deficit of damaged RGCs in glaucomatous eyes is likely to be compensated for. This would explain why visual acuity in glaucoma patients is normal despite the decrease in macular sensitivity. We believe that assessing fixation stability offers an advantage as an objective method that is relatively easy for patients to perform, compared with perimetric tests. The results are promising, and further research is warranted to determine whether this approach could become a useful method for diagnosing and monitoring not only glaucoma but also other neurodegenerative diseases, as suggested in previous studies.

## Figures and Tables

**Table 1:** Demographic data and clinical characteristics of patients.

Parameter	Healthy	POAG		*p*-Value
		Mild	Moderate/Severe	
Number of eyes	23	31	23	
Male:female ratio	5:11	7:13	6:10	0.932
Age, year	67.88 ± 7.61	69.05 ± 8.70	71.06 ± 8.50	0.522
BCVA, Snellen	1.00 ± 0.02	0.99 ± 0.02	0.96 ± 0.08	0.085
IOP, mmHg	18.13 ± 2.07	18.25 ± 2.02	17.66 ± 2.47	0.764
AL, mm	23.32 ± 1.11	23.4 ± 1.06	23.37 ± 0.73	0.965
CCT, µm	532.9 ± 27.20	527.2 ± 40.07	527 ± 55.83	0.574
MP examination time, s	320.84 ± 74.14	307.61 ± 62.79	313.83 ± 78.26	0.387

Data are presented as mean ± one standard deviation or number. Differences among groups were assessed using the χ^2^ test for categorical variables and Kruskal–Wallis test for continuous variables. Abbreviations: POAG, primary open-angle glaucoma; BCVA, best-corrected visual acuity; IOP, intraocular pressure; AL, axial length; CCT, central corneal thickness; MP, microperimetry.

**Table 2:** Differences in structural and functional characteristics between study groups.

Parameter	Healthy	POAG		*p*-Value
		Mild	Moderate/Severe	
GCC average, µm	98.17 ± 9.89	83.32 ± 10.02	72.22 ± 11.38	<0.001
pRNFL global, µm	101.74 ± 10.06	79.42 ± 13.82	61.87 ± 14.12	<0.001
SVP whole VD, %	50.09 ± 2.50	43.47 ± 5.11	39.01 ± 6.32	<0.001
DVP whole VD, %	50.17 ± 3.71	47.94 ± 5.64	47.06 ± 5.45	0.164
DVP/SVP whole VD ratio	0.99 ± 0.08	1.11 ± 0.13	1.21 ± 0.13	<0.001
pRPC VD, %	51.73 ± 2.56	44.48 ± 6.71	36.60 ± 7.36	<0.001
VFI, %	98.30 ± 1.56	95.81 ± 3.53	63.74 ± 27.52	<0.001
MD, dB	−1.52 ± 1.62	−2.57 ± 1.54	−12.77 ± 7.67	<0.001
PSD, dB	2.25 ± 0.66	2.89 ± 1.61	8.26 ± 3.88	<0.001
AT, dB	26.77 ± 1.34	25.32 ± 2.26	20.15 ± 5.38	<0.001

Data are presented as mean ± one standard deviation. Statistically significant differences (*p* < 0.05) are indicated in bold. The Kruskal–Wallis test was used to compare groups. Abbreviations: POAG, primary open-angle glaucoma; GCC, ganglion cell complex; pRNFL, peripapillary retinal nerve fiber layer; VD, vessel density; SVP, superficial vascular plexus; DVP, deep vascular plexus; pRPC, peripapillary radial peripapillary capillaries; VFI, visual field index; MD, mean deviation; PSD, pattern standard deviation; AT, average threshold.

**Table 3:** Differences in fixation indices among study groups.

Parameter	Healthy	POAG		*p*-Value
		Mild	Moderate/Severe	
BCEA63, °²	**0.74 ± 0.60**	0.97 ± 0.90	1.64 ± 1.57	0.051
BCEA95, °²	2.20 ± 1.80	2.93 ± 2.72	4.51 ± 4.12	**0.042**
H95, °	1.49 ± 0.72	1.89 ± 0.91	2.15 ± 1.07	0.135
V95, °	1.54 ± 0.88	1.63 ± 0.93	2.14 ± 1.29	0.091
P1, %	95.39 ± 4.72	92.97 ± 8.40	89.52 ± 13.02	0.127
P2, %	98.65 ± 2.08	98.84 ± 1.57	97.26 ± 3.52	0.196
Angle, °	−11.37 ± 3.93	1.54 ± 33.82	−8.23 ± 46.28	0.480
H95/V95 ratio	1.16 ± 0.59	1.25 ± 0.38	1.05 ± 0.30	0.198

Data are presented as mean ± one standard deviation. Statistically significant differences (*p* < 0.05) are indicated in bold. The Kruskal–Wallis test was used to compare groups. Abbreviations: POAG, primary open-angle glaucoma; BCEA, bivariate contour ellipse area; H, horizontal; V, vertical.

**Table 4:** Spearman’s rank correlation coefficient measuring the relationship between fixation indices and retinal structural parameters (GCC and pRNFL thickness, pRPC and SVP vessel density) of study groups.

Parameter	GCC	GCC S	GCC I	pRNFL G	pRNFLS	pRNFL I	pRNFL T	pRNFL N	pRPC	pRPC S	pRPCI	SVP
P1	**0.247 (0.031)**	**0.255 (0.025)**	0.216 (0.059)	0.149 (0.196)	**0.231 (0.043)**	0.091 (0.43)	**0.333 (0.003)**	−0.008 (0.946)	0.153 (0.184)	0.169 (0.142)	0.119 (0.302)	**0.299 (0.008)**
P2	0.111 (0.336)	0.114 (0.325)	0.099 (0.392)	0.043 (0.71)	0.113 (0.328)	0.005 (0.966)	**0.234 (0.04)**	−0.128 (0.267)	0.07 (0.544)	0.099 (0.393)	0.031 (0.786)	0.171 (0.136)
BCEA63	**−0.256 (0.025)**	**−0.255 (0.025)**	**−0.239 (0.036)**	−0.172 (0.135)	**−0.265 (0.02)**	−0.116 (0.316)	**−0.325 (0.004)**	−0.029 (0.802)	−0.174 (0.131)	−0.193 (0.092)	−0.141 (0.221)	**−0.323 (0.004)**
BCEA95	**−0.273 (0.016)**	**−0.269 (0.018)**	**−0.255 (0.025)**	−0.183 (0.111)	**−0.278 (0.014)**	-0.136 (0.239)	**−0.327 (0.004)**	−0.028 (0.807)	−0.187 (0.103)	−0.204 (0.075)	−0.156 (0.175)	**−0.337 (0.003)**
H	**−0.232 (0.043)**	−0.222 (0.053)	−0.22 (0.056)	−0.134 (0.249)	−0.221 (0.055)	−0.085 (0.466)	**−0.325 (0.004)**	−0.003 (0.978)	−0.119 (0.305)	−0.137 (0.236)	−0.098 (0.4)	**−0.309 (0.007)**
V	−0.2 (0.084)	−0.2 (0.084)	−0.181 (0.118)	−0.139 (0.23)	**−0.242 (0.035)**	−0.107 (0.359)	−0.208 (0.071)	−0.006 (0.956)	−0.201 (0.082)	−0.213 (0.064)	−0.166 (0.152)	**−0.251 (0.029)**

Statistically significant (*p* < 0.05) coefficient values are indicated in bold. *p*-values are presented in brackets. Abbreviations: BCEA, bivariate contour ellipse area; H, horizontal; V, vertical; GCC, ganglion cell complex; pRNFL, peripapillary retinal nerve fiber layer; pRPC, peripapillary radial peripapillary capillaries; SVP, superficial vascular plexus; G, global; S, superior; I, inferior; T, temporal; N, nasal.

**Table 5:** Spearman’s rank correlation coefficient measuring the relationship between fixation indices and retinal function parameters of study groups.

Parameter	AT	VFI	MD	PSD	BCVA
P1	**0.339** **(0.003)**	0.203 (0.083)	0.076 (0.52)	−0.097 (0.411)	0.191 (0.096)
P2	**0.226** **(0.049)**	0.101 (0.393)	0.036 (0.76)	−0.022 (0.849)	0.090 (0.436)
BCEA63	**−0.370** **(0.001)**	**−0.247** **(0.034)**	−0.131 (0.267)	0.127 (0.282)	**−0.233** **(0.041)**
BCEA95	**−0.384** **(0.001)**	**−0.248** **(0.033)**	−0.126 (0.283)	0.134 (0.255)	−0.213 (0.063)
H95	**−0.302** **(0.008)**	−0.21 (0.075)	−0.092 (0.438)	0.105 (0.376)	−0.178 (0.125)
V95	**−0.287** **(0.012)**	−0.21 (0.075)	−0.101 (0.393)	0.084 (0.482)	−0.163 (0.161)

Statistically significant (*p* < 0.05) coefficients are indicated in bold. *p*-values are presented in brackets. Abbreviations: BCEA, bivariate contour ellipse area; H, horizontal; V, vertical; AT, average threshold; VFI, visual field index; MD, mean deviation; PSD, pattern standard deviation; BCVA, best-corrected visual acuity.

## Data Availability

The data presented in this study are available on request from the corresponding author. The data are not publicly available due to ethical restrictions.

## References

[1] Quigley H.A. (2011). Glaucoma. Lancet.

[2] Bowd C., Zangwill L.M., Berry C.C. (2001). Detecting Early Glaucoma by Assessment of Retinal Nerve Fiber Layer Thickness and Visual Function. Investig. Ophthalmol. Vis. Sci..

[3] Zabel K., Zabel P., Kaluzna M. (2020). Correlation of retinal sensitivity in microperimetry with vascular density in optical coherence tomography angiography in primary open-angle glaucoma. PLoS ONE.

[4] Oztürk F., Yavas G.F., Küsbeci T., Ermis S.S. (2008). A comparison among Humphrey field analyzer, Microperimetry, and Heidelberg Retina Tomograph in the evaluation of macula in primary open angle glaucoma. J. Glaucoma.

[5] Krauzlis R.J., Goffart L., Hafed Z.M. (2017). Neuronal control of fixation and fixational eye movements. Phil. Trans. R. Soc. B.

[6] Culham L., Fitzke F.W., Timberlake G.T. (1993). Assessment of fixation stability in normal subjects and patients using a scanning laser ophthalmoscope. Clin. Vis. Sci..

[7] Rohrschneider K., Becker M., Kruse F.E. (1995). Stability of fixation: Results of fundus-controlled examination using the scanning laser ophthalmoscope. Ger. J. Ophthalmol..

[8] Bellmann C., Feely M., Crossland M.D. (2004). Fixation stability using central and pericentral fixation targets in patients with age-related macular degeneration. Ophthalmology.

[9] Schönbach E.M., Ibrahim M.A., Kong X. (2017). Metrics and Acquisition Modes for Fixation Stability as a Visual Function Biomarker. Investig. Ophthalmol. Vis. Sci..

[10] Kosnik W., Fikre J., Sekuler R. (1986). Visual fixation stability in older adults. Investig. Ophthalmol. Vis. Sci..

[11] Montesano G., Crabb D.P., Jones P.R. (2018). Evidence for alterations in fixational eye movements in glaucoma. BMC Ophthalmol..

[12] Shi Y., Liu M., Wang X. (2013). Fixation behavior in primary open angle glaucoma at early and moderate stage assessed by the MicroPerimeter MP-1. J. Glaucoma.

[13] Kameda T., Tanabe T., Hangai M. (2009). Fixation behavior in advanced stage glaucoma assessed by the MicroPerimeter MP-1. Jpn. J. Ophthalmol..

[14] Tewarie P., Balk L., Costello F., Green A., Martin R., Schippling S., Petzold A. (2012). The OSCAR-IB Consensus Criteria for Retinal OCT Quality Assessment. PLoS ONE.

[15] Midena E., Radin P.P., Pilotto E. (2004). Fixation pattern and macular sensitivity in eyes with subfoveal choroidal neovascularization secondary to age-related macular degeneration. A microperimetry study. Semin. Ophthalmol..

[16] Vujosevic S., Midena E., Pilotto E. (2006). Diabetic macular edema: Correlation between microperimetry and optical coherence tomography findings. Invest. Ophthalmol. Vis. Sci..

[17] Fujii G.Y., de Juan E., Sunness J.S. (2002). Patient selection for macular translocation surgery using the scanning laser ophthalmoscope. Ophthalmology.

[18] Steinman R.M. (1965). Effect of target size, luminance, and color on monocular fixation. J. Opt. Soc. Am..

[19] Morales M.U., Saker S., Wilde C. (2016). Reference clinical database for fixation stability metrics in normal subjects measured with the MAIA microperimeter. Trans. Vis. Sci Technol..

[20] Crossland M.D., Rubin G.S. (2002). The use of an infrared eyetracker to measure fixation stability. Optom Vis. Sci..

[21] Tarita-Nistor L., Gonzalez E.G., Markowitz S.N. (2008). Fixation characteristics of patients with macular degeneration recorded with the mp-1 microperimeter. Retina.

[22] Longhin E., Convento E., Pilotto E. (2013). Static and dynamic retinal fixation stability in microperimetry. Can. J. Ophthalmol..

[23] Crossland M.D., Dunbar H.M., Rubin G.S. (2009). Fixation stability measurement using the MP1 microperimeter. Retina.

[24] Gil-Casas A., Llorens D.P.P., Molina-Martin A. (2021). Ocular fixation and macular integrity by microperimetry in multiple sclerosis. Graefe’s Arch. Clin. Exp. Ophthalmol..

[25] Quigley H.A. (1999). Neuronal death in glaucoma. Prog. Retin Eye Res..

[26] Weber A.J., Harman C.D. (2005). Structure-function relations of parasol cells in the normal and glaucomatous primate retina. Investig. Ophthalmol. Vis. Sci..

[27] Gupta N., Yücel Y.H. (2007). Glaucoma as a neurodegenerative disease. Curr. Opin. Ophthalmol..

[28] Anderson T.J., MacAskill M.R. (2013). Eye movements in patients with neurodegenerative disorders. Nat. Rev. Neurol..

[29] Mallery R.M., Poolman P., Thurtell M.J. (2018). Visual fixation instability in multiple sclerosis measured using SLO-OCT. Investig. Ophthalmol. Vis. Sci..

[30] Medeiros F.A., Weinreb R.N. (2002). Medical Backgrounders: Glaucoma. Drugs Today.

[31] Hong Y.J., Shin D.H., Ahn B.H., McCarty B. (1995). Intraocular Pressure after a Two-Week Washout Following Long-Term Timolol or Levobunolol. J. Ocul. Pharmacol. Ther..

[32] Stewart W.C., Holmes K.T., Johnson M.A. (2001). Washout Periods for Brimonidine 0.2% and Latanoprost 0.005%. Am. J. Ophthalmol..

